# A comprehensive evaluation of imaging features in pediatric spinal gliomas and their value in predicting tumor grade and histology

**DOI:** 10.1007/s00234-024-03395-y

**Published:** 2024-06-20

**Authors:** Carmen Rosa Cerron-Vela, Fabrício Guimarães Gonçalves, Luis Octavio Tierradentro-García, Angela N Viaene, Wondwossen Lerebo, Savvas Andronikou

**Affiliations:** 1 Department of Radiology, Children’s Hospital of Philadelphia, University of Pennsylvania, Philadelphia, PA USA; 2 Department of Pathology and Laboratory Medicine, Children’s Hospital of Philadelphia, University of Pennsylvania, Philadelphia, PA United States; 3 Department of Radiology, Children’s of Alabama, Birmingham, USA

**Keywords:** Pediatric spinal cord glioma, Low-grade glioma, High-grade glioma, Spinal cord astrocytomas, Ependymoma

## Abstract

**Purpose:**

Pediatric spinal cord gliomas (PSGs) are rare in children and few reports detail their imaging features. We tested the association of tumoral grade with imaging features and proposed a novel approach to categorize post-contrast enhancement patterns in PSGs.

**Methods:**

This single-center, retrospective study included patients <21 years of age with preoperative spinal MRI and confirmed pathological diagnosis of PSG from 2000-2022. Tumors were classified using the 5th edition of the WHO CNS Tumors Classification. Two radiologists reviewed multiple imaging features, and classified enhancement patterns using a novel approach. Fisher's exact test determined associations between imaging and histological features.

**Results:**

Forty-one PSGs were reviewed. Thirty-four were intramedullary, and seven were extramedullary. Pilocytic astrocytoma was the most common tumor (39.02%). Pain and weakness were the most prevalent symptoms. Seven patients (17.07%) died. Cyst, syringomyelia, and leptomeningeal enhancement were associated with tumor grade. Widening of the spinal canal was observed only in low-grade astrocytomas. There was a significant association between tumor grade and contrast enhancement pattern. Specifically, low-grade PSGs were more likely to exhibit type 1A enhancement (mass-like, with well-defined enhancing margins) and less likely to exhibit type 1B enhancement (mass-like, with ill-defined enhancing margins).

**Conclusion:**

PSGs display overlapping imaging features, making grade differentiation challenging based solely on imaging. The correlation between tumor grade and contrast enhancement patterns suggests a potential diagnostic avenue, requiring further validation with larger, multicenter studies. Furthermore, Low-grade PSGs display cysts and syringomyelia more frequently, and leptomeningeal enhancement is less common.

## Introduction

The family of gliomas, glioneuronal tumors, and neuronal tumors is the most common and diverse group of tumors affecting the Central Nervous System (CNS), as classified in the 2021 5th World Health Organization Classification of CNS Tumors (WHO5). This category includes tumors with glial differentiation (such as adult-type diffuse gliomas, pediatric-type diffuse low-grade gliomas, pediatric-type diffuse high-grade gliomas, and circumscribed astrocytic gliomas), tumors containing both neoplastic glia and neurons (glioneuronal tumors), and those thought to arise from ependymal cells (ependymomas, including spinal ependymomas and myxopapillary ependymomas) [1].

In the pediatric spine, tumors with glial differentiation are the most common, particularly those with astrocytic features and those originating from ependymal cells [2, 3]. Pediatric spinal gliomas (PSGs) can manifest as intramedullary tumors within the spinal cord, including those in the conus medullaris. They may also originate from ependymal cells surrounding the spinal cord, within the central canal, along the cauda equina nerve roots, and in the surrounding filum terminale.

Spinal astrocytomas are generally more common in children than adults, with most being low-grade pilocytic astrocytomas (PA) [3]. Conversely, ependymomas are more prevalent in adults than children [4]. Although gross total resection is generally considered the standard for long-term local control in cases of spinal cord tumors, it carries an elevated risk of post-surgical neurological deficits, particularly in patients with high-grade tumors [5]. Magnetic resonance imaging (MRI) is crucial for effective surgical planning and postoperative counseling, as it is considered the gold standard non-invasive method for the preoperative diagnosis of spinal cord lesions [5–7].

Due to the rarity of these tumors, only a few reports have been published about them, and even fewer provide information on their imaging features for preoperative diagnostic prediction. Understanding the imaging features of the different types and grades of PSGs can provide insights into their nature and behavior, ultimately leading to improved patient outcomes. Therefore, we aim to assess various imaging features and clinical information to determine their association with tumor grade and histological diagnosis in PSGs.

## Methods

### Study design and inclusion criteria

This single-center, retrospective study was reviewed and approved by our institutional review board. A waiver for documentation of informed consent was obtained. We searched our pathology and radiology databases (mPower by Nuance Communications Inc., Burlington, MA, and Illuminate InSight, Overland Park, KA) for biopsy-proven PSGs from 2000-2022. We obtained demographic data from an electronic chart system (Epic Systems Corp., Verona, WI). Inclusion criteria were age at diagnosis below 21 years, preoperative MRI, and histological diagnosis of PSGs. The exclusion criteria included tumors not centered in the spinal canal (i.e., tumors centered within the brainstem and partially involving the spinal cord), brain involvement, disseminated leptomeningeal involvement without obvious primary lesion, prior history of radiation or spinal cord surgery, and secondary PSGs.

### Imaging data acquisition

All patients underwent spine MRI either 1.5 Tesla (T) or 3.0 T (Siemens Medical Solutions, Erlangen, Germany). The slice thickness varied from 2.5 to 4 mm with an interslice gap from 0 to 3 mm. All patients had sagittal and axial T1- and T2-weighted imaging (T1WI and T2WI) of the entire spine. Contrast-enhanced images were obtained using either sagittal T1 fluid-attenuated inversion recovery (FLAIR) or T1 Turbo spin echo (TSE) with fat saturation. Additionally, sagittal diffusion-weighted imaging (DWI) and axial gradient echo (GRE) images were performed in some patients and were evaluated when available.

### Image analysis

All imaging was retrospectively reviewed individually by a pediatric radiologist (with four years of experience) and a pediatric neuroradiologist (with twelve years of experience), who were blinded to histopathological data. Discrepancies were reviewed in consensus. The following imaging features were recorded:

1. Tumor location: Cervical, thoracic, lumbar, or conus medullaris, based on the largest portion of the tumor.
2. Tumor anatomic location: Within the spinal canal. Intramedullary or extramedullary.
3. Tumor axial location: Centric or eccentric in relation to the spinal cord. Only assessed in intramedullary tumors.
4. Morphology: Solid (without cysts or necrosis), predominantly solid (solid components making up 80% of the tumor), mixed solid and cystic (without predominance of either component), or predominantly cystic (solid components making up less than 20% of the tumor).
5. Lesion size: Tumor longitudinal extent in the sagittal plane, measured relative to the number of adjacent vertebral bodies.
6. Margins: Well-defined or ill-defined, based on the subjective visual outline of the tumor borders and the interface between the tumor and normal cord parenchyma.
7. Cysts: Well-circumscribed area with thin, non-enhancing, or regular linear enhancing walls, smooth regular margins, central T2WI hyperintensity, and T1WI hypointensity matching that of cerebrospinal fluid (CSF).
8. Hemorrhage: Non-enhancing areas with T1WI hyperintensity and T2WI hypointensity. On GRE imaging, areas with low signal.
9. Cap sign: Peripheral T1 hyperintensity and T2 hypointensity due to hemosiderin deposition.
10. Syringomyelia: Defined as a non-enhancing cystic dilatation of the central canal.
11. Edema: T2WI hyperintensity within the spinal cord adjacent to the tumor.
12. T1WI and T2WI signal: High or low signal intensity based on the signal characteristics of the solid tumoral component compared to the spinal cord.
13. Diffusion characteristics: Qualitative diffusion analysis was performed when available. Lesions were classified into diffusion-restricted and non-restricted.
14. Enhancement pattern: Type 1 (type 1a, type 1b) and Type 2. Based on the enhancement of the solid component in the sagittal plane:

#### Type 1, nodular or mass-like enhancement

Defined as an expansile appearance of the enhancing component, varying from nodular, multinodular/lobulated, or long and “sausage-like.” Type 1 is further divided into 1a (crisp, well-defined, smooth enhancing margins) and 1b (shaggy, irregular, ill-defined enhancing margins, with subcategories of central enhancement and central non-enhancement).

#### Type 2, patchy enhancement

Defined as an ill-defined enhancement pattern without a nodular or mass-like appearance.

#### Type 3, no enhancement

Defined as the absence of tumor enhancement after contrast administration.

**Fig. 1:**
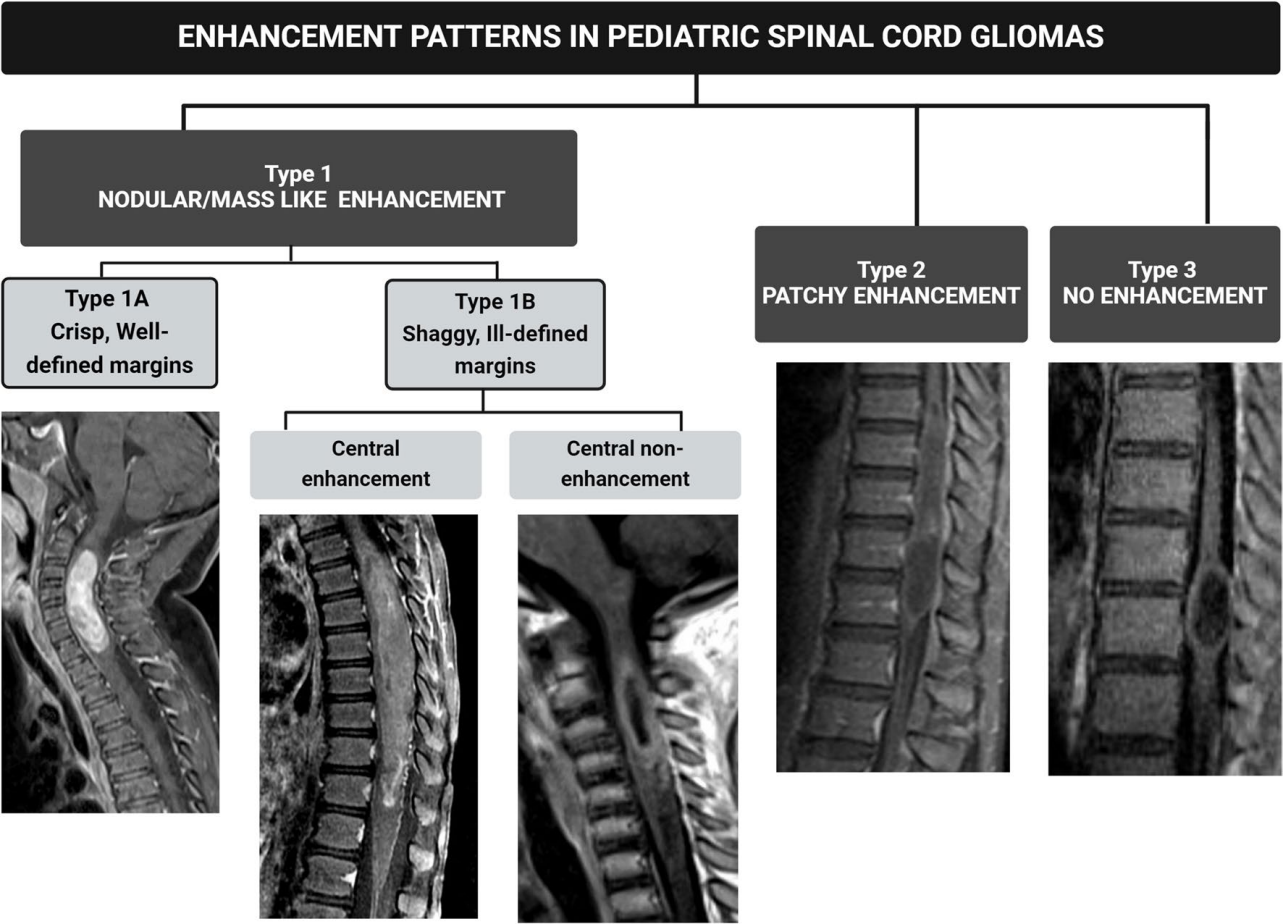
Proposed classification of enhancement patterns in pediatric spinal cord gliomas

**Fig. 2:**
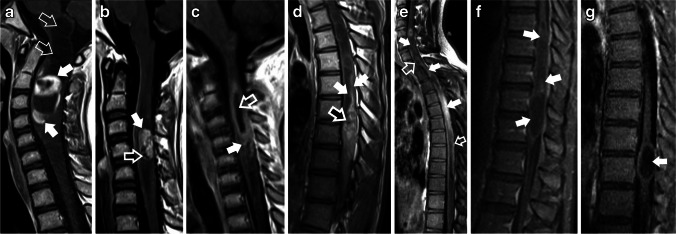
(**a - g**) Examples of pediatric spinal gliomas illustrating the enhancement patterns identified in the proposed classification system. **a**) Type 1A enhancement in a Pilocytic astrocytoma in a 10-year-old girl presenting with a one-month history of neck pain and recent right arm weakness. A contrast-enhanced sagittal T1-weighted image shows an expansile, intramedullary solid and cystic mass in the cervical cord at levels C1-C3. This mass exhibits a nodular/mass-like enhancement pattern with crisp, well-defined margins (solid white arrows). Additionally, a syrinx is present at the mass superior pole, extending into the lower medulla, and exhibits no enhancement (hollow white arrows). **b**) Type 1A enhancement in a Diffuse pediatric-type High-Grade glioma, H3-wildtype, and IDH-wildtype in a 16-year-old female with a two-week history of progressive left-sided weakness. Sagittal contrast-enhanced T1-weighted image shows a predominantly solid intramedullary tumor in the cervical cord at levels C3 to C4 (solid white arrow). This tumor measures 20 mm in length, displays nodular/mass-like enhancement and well-defined borders, and contains a few cystic/necrotic areas (hollow white arrow). **c**) Type 1B enhancement is seen in a 7-year-old girl with a 5-day history of neck pain and stiffness, diagnosed with a Diffuse midline glioma with H3 K27 alteration. Sagittal contrast-enhanced T1-weighted image shows a 40 mm long expansile intramedullary tumor within the cervical cord, extending from C2 to C4. This tumor exhibits nodular/mass-like enhancement with ill-defined/shaggy borders (solid white arrow), alongside a central area of non-enhancement, (hollow white arrow). **d**) Type 1B enhancement in a Pilocytic astrocytoma in a 19-year-old male with a 10-month history of progressive sensory loss and weakness in the lower extremities. Sagittal contrast-enhanced T1-weighted image reveals a 130 mm long expansile solid intramedullary tumor within the thoracic cord, extending from T6 to T10. The tumor displays nodular/mass-like central enhancement (hollow white arrow) and ill-defined, shaggy borders (solid white arrows). **e**) Type 2 enhancement in a High-grade glioma NOS, diagnosed in a 19-year-old male with a two-month history of left-sided weakness. Sagittal contrast-enhanced T1-weighted image shows a long expansile intramedullary mass extending from C4 to T11. The enhancement is ill-defined, lacking a nodular or mass-like appearance (solid white arrows). Additionally, diffuse leptomeningeal enhancement is noted (hollow white arrows). f) Type 2 enhancement in a Pilocytic astrocytoma in a 15-year-old boy presenting with a 7-month history of progressive weakness in the left lower extremity and an unsteady gait. Sagittal contrast-enhanced T1-weighted image reveals a large, infiltrative intramedullary lesion within the thoracic cord. This lesion spans from T8 to T12 and measures 100 mm in length. It is characterized by ill-defined, patchy enhancement and the absence of a well-defined nodule, as indicated by the solid white arrows. **g**) Type 3 enhancement in a Pilocytic astrocytoma in a 17-year-old male with a history of back pain. Sagittal contrast-enhanced fat-saturated T1 fluid-attenuated inversion recovery image reveals a well-defined, oval-shaped intramedullary mass located in the thoracic spine at the T10-T11 level. The mass, measuring 30 mm in length, is notable for its lack of enhancement (solid white arrow)

These patterns were mutually exclusive. The proposed classification system in Fig. 1 outlines distinct categories of intraspinal astrocytic tumors based on their enhancement patterns. Some examples are depicted in Fig. 2.

### Pathological features

Surgically sampled spinal tumors were reviewed by a board-certified pediatric neuropathologist blinded to clinical and radiologic data. Molecular testing results from the original clinical workup were also reviewed when available. Tumors were re-classified according to the WHO5.

### Data analysis

Statistical analysis was conducted using STATA (version 17). Descriptive statistics were used to summarize all variables. Qualitative data are presented using frequency and percentages; parametric quantitative data using mean and standard deviation (SD); and non-parametric data using median and interquartile range (IQR). Fisher’s exact test was used to determine associations between imaging features and histological classification for histological diagnosis and tumor grade. A p-value < 0.05 was considered statistically significant. Associations were performed in four different scenarios: a comparison of imaging features of intramedullary spinal cord astrocytomas and intramedullary spinal cord ependymomas; a comparison of imaging features between high-grade intramedullary spinal cord gliomas (including both astrocytomas and ependymomas) and low-grade intramedullary spinal cord gliomas; a comparison of imaging features between of high-grade spinal cord astrocytomas and low-grade spinal cord astrocytomas only; and a comparison of imaging features of high-grade spinal cord ependymomas and low-grade spinal cord ependymomas only. This final analysis included both intramedullary and extramedullary tumors.

## Results

### Demographics and clinical data

Forty-one children were diagnosed with PSGs and underwent preoperative MRI scans; 46.34% (*n=*19) were girls. The mean age at diagnosis was 10 (SD 5.98) years. In most patients, the onset of symptoms occurred less than three months before diagnosis (63.41%, *n=*26). Pain (63.41%, *n=*26) and weakness (53.66%, *n=*22) were the most frequent symptoms. Seven patients (17.07%) died. Six of them were high-grade astrocytic tumors (WHO 4) and died within 3-13 months of diagnosis. One patient died of pilocytic astrocytoma malignant transformation after 147 months with widespread brain and leptomeningeal metastasis. No deaths were found in patients with high-grade ependymomas (WHO 3). A summary of clinical findings is presented in Table 1.

**Table 1:** Demographic and clinical data of 41 pediatric primary spinal cord gliomas at one hospital in the USA over a 20-year period

Demographic and clinical data	
Age	
0 to 3	6 (14.63)
4 to 12	21 (51.22)
13 to 18	14 (34.15)
Sex	
Male	22 (53.66)
Female	19 (46.34)
Symptoms	
Weakness	22 (53.66)
Pain	26 (63.41)
Numbness/tingling	7 (17.07)
Paralysis	2 (4.88)
Duration of Symptoms	
< 3 months	26 (63.41)
> 3 months	8 (19.51)
Not given	7 (19.51)
Deceased	7 (17.07)

Values between brackets represent percentages

### Pathological findings

Among the 41 cases, 65.85% (*n=* 27) were astrocytomas and 34.15% (*n=*14) were ependymomas. Overall, pilocytic astrocytoma (PA) was the most common tumor (39.02%, *n=*16) followed by spinal ependymoma (24.39%, *n=*10). The most common high-grade astrocytoma was diffuse pediatric-type high-grade glioma, H3-wildtype, and IDH-wildtype (DPHGG) (9.76%, *n=*4). Similarly, the most prevalent high-grade ependymal tumor was spinal ependymoma, also constituting 9.76% (*n=*4). Pathology findings are summarized in Table 2.

**Table 2:** Pathology of 41 pediatric primary spinal cord gliomas at one hospital in the USA over a 20-year period

Pathology	
WHO grade	
Low grade (WHO I and II)	13 (31.71)
High grade (WHO III and IV)	28 (68.29)
Integrated diagnosis	
Diffuse midline glioma H3 K27M-altered	3 (7.32)
High-grade glioma, NOS	3 (7.32)
Diffuse pediatric type high-grade glioma, H3-wildtype and IDH-wildtype	4 (9.76)
Pilocytic astrocytoma	16 (39.02)
Low-grade gliomas, NEC	1 (2.44))
Myxopapillary ependymoma	4 (9.76)
Spinal ependymoma	10 (24.39)

### Imaging findings

All astrocytic tumors were located intramedullary, accounting for 100% of the cases (*n=*27). Ependymomas, on the other hand, were found both intramedullary (50%, *n=*7) and extramedullary (50%, *n=*7). Most astrocytomas exhibited hypointensity on T1WI and hyperintensity on T2WI. In contrast, ependymomas also displayed isointense signals on T1WI (35.71%, *n=*5) and T2WI (35.71%, *n=*5). The most common tumor location was the cervical spine (35.0%, *n=*14), followed by the thoracic spine (27.50%, *n=*11). Myxopapillary ependymomas were all extramedullary, and three of which were seen adjacent the conus medullaris. Intramedullary ependymomas were not seen in this location. One spinal ependymoma was at the dural sac terminus, within the sacral region.

The most common tumor morphology observed was solid, accounting for 39.02% (*n=*16) of cases, followed by predominantly solid morphology seen in 34.15% (*n=*14). Edema was present in 27 patients (65.85%), and syringomyelia was identified in 14 patients (34.15%). Tumor margins were well-defined in 68.85% (*n=*27) of cases. Hemorrhage was detected in 15 cases (36.59%), and the 'cap sign' was found in 5 cases (12.20%). Spinal canal widening was present in 48.78% of patients (*n=*20), and scoliosis was observed in 14.63% (*n=*6). Our study found that when scoliosis was present, it was associated with spinal canal widening and an underlying low-grade astrocytoma.

Contrast enhancement was present in 92.68% of cases (*n=*38). Based on our newly proposed enhancement classification system, Type 1a was the most common pattern of enhancement accounting for 65.85% (*n=*27) of cases, followed by Type 1b (17.07%, *n=*7), Type 2 (9.76%, *n=*4), and Type 3 (7.32%, *n=*3). Leptomeningeal enhancement was present in 31.71% of cases (*n=*13). Of the tumors with available diffusion sequences, all but one spinal ependymoma (WHO 3) showed no restricted diffusion. All evaluated imaging features are summarized in Table 3.

**Table 3:** Location, extension, situation, axial location, T1/T2 signal, margins, morphology, and presence of cysts, edema, syringomyelia, hemorrhage, cap sign, enhancement (including pattern and leptomeningeal), spinal canal widening and scoliosis of pediatric spinal gliomas

Imaging features	
Location*	
Cervical	14 (35.00)
Cervicothoracic	6 (15.00)
Thoracic	11(27.50)
Thoracolumbar	4 (10.00)
Conus	5(12.50)
Extension	
< 3 VB	17 (41.46)
> 3 VB	24 (58.54)
Anatomic location	
Intramedullary	34 (82.93)
Extramedullary **	7 (17.07)
Axial location ***	
Eccentric	11 (32.35)
Centric	23 (67.65)
T1 signal	
High	
Low	36 (87.80)
Iso	5 (12.20)
T2 signal	
High	36 (87.80)
Low	
Iso	5 (12.20)
Margins	
Well-defined	27 (68.85)
Ill-defined	14 (34.15)
Morphology	
Solid (with no cysts or necrosis)	16 (39.02)
Predominantly solid (>80%)	14 (34.15)
Mixed solid and cystic/necrotic	10 (24.39)
Cystic/necrotic (>80%)	1 (2.44)
Cysts	
Yes	16 (36.59)
No	26 (63.41)
Edema	
Yes	27 (65.85)
No	14 (34.15)
Syringomyelia	
Yes	14 (34.15)
No	27 (65.85)
Hemorrhage	
Yes	15 (36.59)
No	26 (63.41)
Cap sign	
Yes	5 (12.20)
No	36 (87.80)
Enhancement	
Yes	38 (92.68)
No	3 (7.32)
Pattern 1a	27 (65.85)
Pattern 1b	7 (17.07)
Pattern 2 (Patchy)	4 (9.76)
Pattern 3 (None)	3 (7.32)
Leptomeningeal enhancement	
Yes	13 (31.71)
No	28 (68.29)
DWI	
Yes	1 (9.09)
No	10 (90.91)
Spinal canal widening	
Yes	20 (48.78)
No	21 (51.22)
Scoliosis	
Yes	6 (14.63)
No	35 (85.27)

*one spinal ependymoma was located in the sacrum

**all extramedullary tumors were myxopapillary ependymomas

***only evaluated in intramedullary tumors

Values between brackets represent percentages

As all extramedullary tumors were identified as ependymomas, the evaluation of imaging features in relation to histology and tumor grade was conducted exclusively for intramedullary tumors. Among the thirty-four intramedullary tumors, comprising ependymomas (*n=*7) and astrocytomas (*n=*27), 23 were classified as low-grade and 11 as high-grade.

**Fig. 3:**
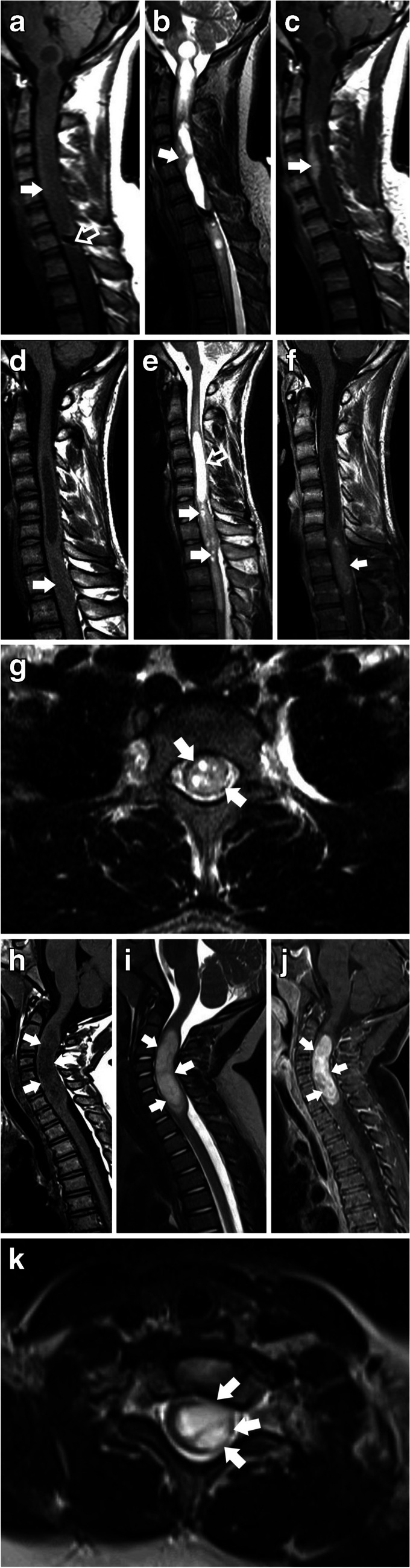
(**a - k**). Examples of Imaging Features in Astrocytomas vs. Ependymomas. (**a** – **c**) Spinal ependymoma WHO 2 in an 18-year-old boy experiencing chronic neck pain, recent onset of weakness in the right hand, decreased deep tendon reflexes in both upper extremities and increased deep tendon reflexes in both lower extremities. **a**) Sagittal T1 TSE imaging depicts a large, expansile lesion characterized by a predominantly isointense signal (solid white arrows). The mass extends from the pontomedullary junction to the inferior T4 vertebral body. A notable feature is the presence of hemosiderin deposits at the level of C7/T1, indicative of the cap sign (hollow white arrow). **b**) Sagittal T2 reveals a predominantly cystic lesion with solid tissue seen along the periphery extending from C3-C5 (solid white arrow). **c**) Sagittal T1 TSE, after contrast administration, results in enhancement of the solid component extending from C3-C5, demonstrating a nodular/mass-like enhancement pattern with ill-defined/shaggy borders (Type 1b) (solid white arrow). (**d** – **g**) Spinal ependymoma WHO 2 in a 17-year-old male with 1 week of leg numbness and weakness. **d**) Sagittal T1 shows an isointense intramedullary expansile lesion extending from C7 to T2 levels and measuring 45mm in length (solid white arrow). **e**) Sagittal T2 shows multiple intrinsic foci of T2 hyperintensity, representing cystic/necrotic changes within the mass (solid white arrows). There is associated syringomyelia extending from C2 to C6 (hollow white arrow). **f**) Sagittal T1 TSE after contrast administration shows a nodular/mass-like enhancement pattern with well-defined/crisp borders (type 1a) (solid white arrow). **g**) Axial T2 shows the mas has a central location within the spinal cord (solid white arrows). (**h** - **k**) Pilocytic astrocytoma in a 3-year-old girl with a 5-week history of left arm weakness. **h**) Sagittal T1 shows a hypointense expansile solid lesion, extending from C2 to C7, measuring 45mm in length (solid white arrows). **i**) Sagittal T2 shows hyperintense signal of the solid component (solid white arrows). **j**) Sagittal T1 fat-saturated MR image, after contrast administration, shows a nodular/mass-like enhancement and well-defined/crisp borders (type 1a) (solid white arrows). **k**) Axial T2 shows a slightly eccentric mass (solid white arrows)

Ependymomas were more likely to show isointense T2 signals than astrocytomas (*p=*0.003). A statistically significant difference was also observed in terms of morphology and tumor histology, with astrocytomas displaying a higher tendency to be entirely solid tumors. (*P=*0.002). (Fig. 3).

**Fig. 4:**
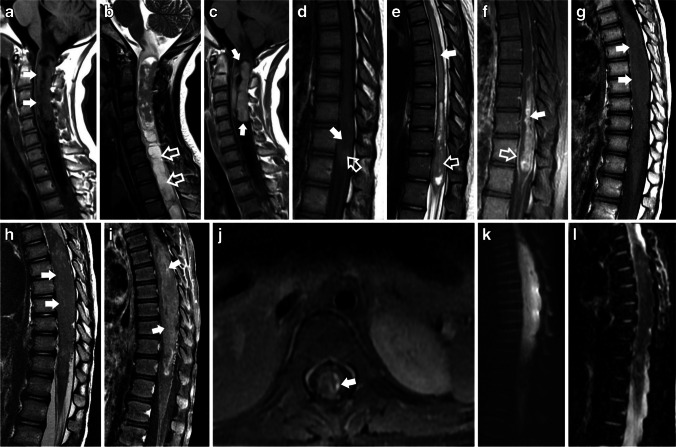
(**a – k**) Examples of the imaging features of high-grade vs. low-grade pediatric spinal gliomas. (**a** – **c**) Pilocytic astrocytoma in an 11-year-old girl, found incidentally. **a**) Sagittal T1 and **b**) T2 show a heterogeneous, solid and cystic/necrotic, expansile mass, extending from the cervico-medullary junction caudally to the C5- C6 level (solid white arrows in a), with an associated extensive caudal syrinx (hollow white arrows in b). **c**) Sagittal T1 FLAIR after contrast administration shows a sausage-shaped tumor with nodular/mass-like enhancement and well-defined/crisp borders (type 1a) (solid white arrows). (**d** – **f**) Diffuse pediatric-type High-grade glioma, H3-wildtype, and IDH-wildtype in a 13-year-old girl, who presented with a one-year history of back pain and headaches, and 3 weeks of acute worsening and loss of ability to walk. **d**) Sagittal T1-weighted MRI image highlighting an ill-defined, expansile lesion in the distal spinal cord. This lesion displays areas of slightly hyperintense signal (hollow white arrow), with a central zone which is isointense to the normal spinal cord (solid white arrow). In conjunction with the sagittal T2 FSE findings (e), the T1 hyperintense signal areas (hollow white arrows) are suggestive of hyperproteinaceous fluid. **e**) Sagittal T2 FSE shows the solid-cystic/necrotic morphology of the lesion (hollow white arrow), associated with extensive edema extending from T4 to T7 (solid white arrow). The mass is centered in the thoracic cord and extends from T8 to T12. **f**) Sagittal T1 with fat saturation after contrast administration, shows a nodular/mass-like enhancing pattern with ill-defined/shaggy borders (Type 1b) and central enhancement (solid white arrow). Leptomeningeal enhancement is also present (hollow white arrow). (**g** – **l**) Spinal ependymoma (WHO 3) in a 3-year-old boy with initial presentation of progressive lower limb weakness that resulted in paraplegia, over a 3-week course. **g**) Sagittal T1 and **h**) Sagittal T2 show a mostly T1/T2 isointense, expansile mass, extending from T4 to T12 (solid white arrows in g and h). **i**) Sagittal T1 with fat saturation after contrast administration demonstrates a nodular/mass-like enhancement and ill-defined/shaggy borders (Type 1b) (solid white arrows). **j**) Axial T1 post-contrast administration with fat saturation shows nodular leptomeningeal enhancement in the left side of the lesion (solid white arrow). **k**) DWI shows high signal intensity which corresponds to l) Low signal intensity on the ADC map, in keeping with restricted diffusion

The analysis of imaging characteristics between intramedullary primary spinal gliomas (PSG) and tumor grade, revealed statistically significant differences in the presence of cysts (*p=* 0.01), syringomyelia (*p=* 0.02), and leptomeningeal enhancement (*p=*0.007). Low-grade PSGs were more likely to display cysts and syringomyelia, while leptomeningeal enhancement was less likely. A statistically significant association was found between tumor grade and contrast enhancement patterns. Low-grade PSG were more likely to exhibit type a 1A pattern of enhancement (*p=*0.030) and less likely to exhibit a type 1B pattern of enhancement. (*P*=0.024). (Fig. 4).

**Fig. 5:**
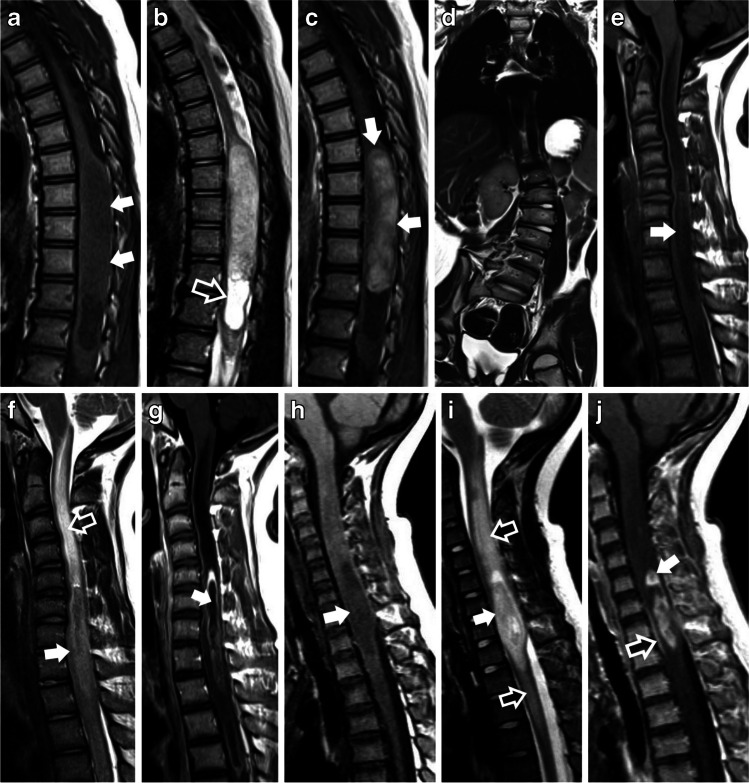
(**a – j**) Examples of High-grade vs Low-grade astrocytomas. (**a** – **d**) Pilocytic astrocytoma in a 4-year-old boy who presented with a 2-month history of progressive right lower leg weakness and gait difficulty. **a**) Sagittal T1 and **b**) Sagittal T2 show an expansile, solid, intramedullary mass in the thoracic cord, extending from T7 to T12 (solid white arrow in a), with an associated caudal syrinx (white hollow arrow in b) and spinal canal widening. **c**) Sagittal T1 after contrast administration demonstrates a sausage-shaped tumor with nodular/mass-like enhancement and well-defined/crisp borders (Type 1A) (solid white arrow). d) Coronal T1 composition of the entire spine shows marked levo-convex scoliosis of the thoracolumbar spine from T10-L5. (**e** – **g**) Diffuse midline glioma H3 K27-altered in a 13-year-old girl who presented with 3 weeks of progressive weakness in her arms and legs. **e**) Sagittal T1 and **f**) Sagittal T2 TSE images show an expansile, ill-defined, intramedullary mass, extending from C5 to T2 (solid white arrow). The mass is associated with edema which extends rostrally to the lower medulla (white hollow arrows in f). **g**) T1 FLAIR after contrast administration shows a nodular/mass-like enhancing pattern with ill-defined/shaggy borders (type1b) and central non-enhancement (solid white arrow). (**h** – **j**) Diffuse pediatric-type High-grade glioma in a 5-year-old male with a rapid onset of right-hand weakness with right-sided upper back and shoulder pain. **h**) Sagittal T1 and **i**) Sagittal T2 TSE show an expansile, well-defined, solid-cystic/necrotic, intramedullary mass in the cervico-thoracic cord, extending from C5 to the upper endplate of T2 (solid white arrow in h and i). There is associated edema extending rostrally to the lower medulla and caudally to the level of T5 (white hollow arrows). **j**) Sagittal T1 TSE, obtained after contrast administration, shows a nodular/mass-like enhancing pattern with a lobulated appearance, and well-defined/crisp borders (type 1a) (solid white arrow). Note the associated leptomeningeal enhancement (hollow white arrow)

When specifically analyzing the imaging features of spinal cord astrocytomas with tumor grade, cysts (*p=*0.042) and leptomeningeal enhancement (*p=*0.025) were also associated with tumor grade. Spinal canal widening (*p=*0.018) and edema (*p=*0.026), were also significant in this group. (Fig. 5). A statistically significant difference was again demonstrated in tumor-enhancing patterns. Low-grade astrocytomas were less likely to present a Type 1b pattern (*p=*0.047). Finally, when evaluating ependymomas alone, there was no statistically significant association between imaging features and tumor grade. Fisher’s exact test values are summarized in Table 4.

**Table 4:** Comparison of MRI Imaging features vs. histological features

Variable	Intramedullary Spinal Gliomas		*P* value	Intramedullary Spinal Gliomas		*P* value	Spinal Astrocytomas		*P* value	Spinal ependymomas		*P* value
	Astrocytoma	Ependymoma		High grade	Low grade		High grade	Low grade		High grade	Low grade	
Location*			0.557			0.749						0.517
Cervical	10 (37.04)	3 (42.86)		4 (36.36)	9 (39.13)		4 (40)	6 (35.29)	0.453	3 (30.00)	1 (33.33)	
Cervico-thoracic	4 (14.81)	2 (38.57)		3 (27.27)	3 (13.04)		3 (30.00)	1 (5.88)		2 (20.00)	0 (0)	
Thoracic	10 (37.04)	1 (14.29)		4 (36.36)	7 (30.43)		3 (30.00)	7 (41.18)		0 (0)	1 (33.33)	
Thoracolumbar	1 (3.70)	1 (14.29)		0 (0)	2 (8.70)		0 (0)	1 (5.88)		2 (66.67)	1 (33.33)	
Conus	2 (7.41)	0 (0)		0 (0)	2 (8.70)		0 (0)	2 (11.76)		3 (30.00)	0 (0)	
Extension			0.097			1			0.683			0.055
< 3 vertebral bodies	9 (33.33)	5 (71.43)		4 (36.36)	10 (43.48)		4 (40.00)	5 (29.41)		8 (72.73)	0 (0)	
> 3 vertebral bodies	18 (66.67)	2 (28.57)		7 (63.64)	13 (56.52)		6 (60.00)	12 (70.59)		3 (27.27)	3 (100.00)	
Anatomic location												1
Intramedullary										6 (54.55)	1 (33.33)	
Extramedullary										5 (45.45)	2 (66.67)	
Axial location						1			1			
Eccentric	11 (40.74)	0 (0)	0.069	4 (36.36)	7 (30.43)		4 (40.00)	7 (41.18)		0 (0)	0 (0)	
Centric	16 (59.26)	7 (100.00)		7 (63.64)	16 (69.57)		6 (60.00)	10 (58.82)		6 (100.00)	1 (100.00)	
Margins			1			0.458			0.224			1
Well defined	16 (59.26)	4 (57.14)		5 (45.45)	15 (65.22)		4 (40.00)	12 (70.59)		8 (72.73)	3 (100.00)	
Ill defined	11 (40.74)	3 (42.86)		6 (54.55)	8 (34.78)		6 (60.00)	5 (29.41)		3 (27.27)	0 (0)	
T1 signal			0.1			0.638			1			1
High	0 (0)	0 (0)		0 (0)	0 (0)		0 (0)	0 (0)		0 (0)	0 (0)	
Low	25 (92.59)	3 (42.86)		10 (90.91)	18 (78.26)		9 (90.00)	16 (94.12)		3 (75.00)	6 (60.00)	
Iso	2 (7.41)	4 (57.14)		1 (9.09)	2 (21.74)		1 (10.00)	1 (5.88)		1 (25.00)	4 (40.00)	
T2 signal			0.003			1			1			0.58
High	26 (96.30)	3 (42.86)		10 (90.91)	20 (86.96)		10 (100.00)	16 (94.12)		2 (50.00)	7 (70.00)	
Low	0 (0)	0 (0)		0 (0)	0 (0)		0 (0)	0 (0)		0 (0)	0 (0)	
Iso	1 (3.70)	4 (57.14)		1 (9.09)	3 (13.04)		0 (0)	1 (5.88)		2 (50.00)	3 (30.00)	
Morphology						0.179			0.164			1
Solid (with no cysts or necrosis)	13 (48.15)	0 (0)	0.002	6 (54.55)	7 (30.43)		6 (60.00)	7 (41.18)		2 (18.18)	1 (33.33)	
Predominantly solid (>80%)	5 (18.52)	6 (85.71)		4 (36.36)	7 (30.43)		3 (30.00)	2 (11.76)		7 (63.64)	2 (66.67)	
Mixed solid and cystic/necrotic	9 (33.3)	1 (10)		1 (9.09)	9 (39.13)		1 (10.00)	8 (47.06)		2 (18.18)	0 (0)	
Cysts			0.41			0.011			0.042			1
Yes	10 (37.04)	4 (57.14)		1 (9.09)	13 (56.52)		1 (10.00)	9 (52.94)		4 (36.36)	1 (33.33)	
No	17 (62.96)	3(42.86)		10 (90.91)	10 (43.48)		9 (90.00)	8 (47.06)		7 (63.64)	2 (66.67)	
Edema			0.3			0.069			0.026			1
Yes	20 (74.07)	7 (100)		11 (100.00)	16 (69.57)		10 (100.00)	10 (58.82)		6 (54.55)	1 (33.33)	
No	7 (25.93)	0 (0)		0 (0)	7 (30.43)		0 (0)	7 (41.18)		5 (45.45)	2 (66.67)	
Syringomyelia			0.387			0.024			0.091			1
Yes	9 (33.33)	4 (57.14)		1 (9.09)	12 (52.17)		1 (10.00)	8 (47.06)		4 (36.36)	1 (33.33)	
No	18 (66.67)	3 (42.86)		10 (90.09)	11 (47.83)		9 (90.00)	9 (52.94)		7 (63.64)	2 (66.67)	
Hemorrhage			0.667			0.459			0.683			0.538
Yes	9 (33.33)	3 (42.86)		5 (45.45)	7 (30.43)		4 (40.00)	5 (29.41)		4 (36.36)	2 (66.67)	
No	18 (66.67)	4 (57.14)		6 (54.55)	16 (69.57)		6 (60.00)	12 (70.59)		7 (63.64)	1 (33.33)	
Cap sign			0.101			0.535			1			0.505
Yes	1 (3.70)	2 (28.57)		0 (0)	3 (13.04)		0 (0)	1 (100)		4 (36.36)	0 (0)	
No	26 (96.30)	5 (71.43)		11 (100.00)	20 (86.96)		10 (100)	16 (61.54)		7 (63.64)	3 (100.00)	
Enhancement			1			0.535			0.159			
Yes	24 (88.89)	7 (100)		11 (100.00)	20 (86.96)		10 (100.00)	14 (82.35)		11 (100.00)	3 (100.00)	
No	3 (11.11)	0 (0)		0 (0)	3 (13.04)		0 (0)	3 (17.65)		0 (0)	0 (0)	
Pattern 1a			1			0.03			0.057			1
Yes	15 (55.56)	4 (57.14)		3 (27.27)	16 (69.57)		3 (30.00)	12 (70.59)		3 (75.00)	8 (80.00)	
No	12 (44.44)	3 (42.86)		8 (72.73)	7 (30.43)		7 (70.00)	5 (29.41)		1 (25.00)	2 (20.00)	
Pattern 1b			0.6			0.024			0.047			0.5
Yes	5 (18.52)	2 (28.57)		5 (45.45)	2 (8.70)		4 (40.00)	1 (5.88)		1 (25.00)	1 (10.00)	
No	22 (81.48)	5 (71.43)		6 (54.55)	21 (91.30)		6 (60.00)	16 (94.12)		3(75.00)	9 (90.00)	
Pattern 2 (Patchy)			0.511			0.239			0.128			1
Yes	2 (7.41)	1 (14.29)		2 (18.18)	1 (4.35)		2 (20.00)	0 (0)		1 (9.09)	0 (0)	
No	25 (92.59)	6 (85.71)		9 (81.82)	22 (95.65)		8 (80.00)	17 (100.00)		10(90.91)	3 (100.00)	
Pattern 3 (None)			1			0.535			0.274			
Yes	3 (11.1)	0 (0)		0 (0)	3 (13.04)		0 (0)	3 (100.00)		11 (100.00)	3 (100.00)	
No	24 (88.89)	7 (100.00)		11 (100)	20 (86.96)		10 (100.00)	14 (82.35)		0 (0)	0 (0)	
Leptomeningeal enhancement						0.007			0.025			0.505
Yes	8 (29.63)	0 (0)	0.16	6 (54.55)	2 (8.70)		6 (60.00)	2 (11.76)		3 (27.27)	2 (66.67)	
No	19 (70.37)	7 (100.00)		5 (45.45)	21 (91.30)		4 (40.00)	15 (88.24)		8 (72.73)	1(33.33)	
DWI			0.22			0.333						1
Yes	0 (0)	1 (50)		1 (33.33)	0 (0)		0 (0)	0 (0)		0 (0)	1 (50.00)	
No	7 (100)	1 (50)		2 (66.67)	6 (100.00)		2 (100.00)	5 (100.00)		2 (100.00)	1 (50.00)	
Spinal canal widening			0.405			0.152			0.018			0.538
Yes	14 (51.85)	2 (28.57)		3 (27.27)	13 (56.52)		2 (20.00)	12 (70.59)		4 (36.36)	2 (66.67)	
No	13 (48.15)	5 (71.43)		8 (72.73)	10 (43.48)		8 (80.00)	5 (29.41)		7 (63.64)	1 (33.33)	
Scoliosis			0.306			0.145			0.057			
Yes	6 (22.22)	0 (0)		0 (0)	6 (26.09)		0 (0)	6 (100.00)		0 (0)	0 (0)	
No	21 (77.78)	7 (100.00)		11 (100.00)	17 (73.91)		10 (100.00)	11 (64.71)		11(100.00)	3 (100.00)	

*One case was located in the sacrum

Values between brackets represent percentages

## Discussion

In this study, we evaluated the preoperative MRI imaging features of PSG based on WHO5 [1] exclusively in the pediatric population under 21 years of age.

Intramedullary tumors can present with nonspecific symptoms, posing challenges in accurate diagnosis [8]. The growth of the neoplasm leads to a gradual deformation of the spinal cord, which in turn can cause a range of symptoms, including pain, numbness, sensory deficits, motor weakness, and disturbances in bowel and bladder function [3, 9, 10]. Similarly, tumors with extramedullary components, depending on their location, may produce comparable symptoms [8, 11]. In our study, the most frequently observed clinical symptoms were pain and weakness. This aligns with findings from previous studies [12, 13], where no significant correlation was found between the duration of symptoms and tumor grade. Notably, in the case of astrocytomas, tumor grading emerges as the strongest predictor of survival. It has been established that the prognosis for astrocytoma is generally less favorable compared to ependymoma. [14]. This observation is consistent with the mortality rates reported in our study.

While ependymomas can arise at any spinal cord level, the cervical cord is the most common site for intramedullary ependymomas, in contrast to astrocytomas, which predominantly occur in the thoracic spine [12, 14, 15]. Consistent with previous findings, our research also indicates that about 50% of ependymomas originate from the terminal filum or conus medullaris, and these are predominantly myxopapillary ependymomas [16]. Other authors have suggested that diffuse/fibrillary astrocytoma (WHO 2) is typically observed in the cervical spine, whereas pilocytic astrocytoma (WHO 1) is primarily found in the conus medullaris [17]. Additionally, Cheng et al. [18] found that DMG H3K27-altered tumors are more frequently in the thoracic spine. In our study, we did not observe any significant correlation between tumor grade and its location within the spinal cord. This lack of association could be attributed to our limited sample size and the exclusive focus on a pediatric population.

A well-documented characteristic of astrocytomas and ependymomas is their intramedullary location. Astrocytomas are typically described as eccentric, whereas ependymomas tend to be more centrally located [19]. In line with these descriptions, our study, albeit not reaching statistical significance, indicates that intramedullary eccentric tumors are less likely to be ependymomas. Originating from the ependymal canal, ependymomas exhibit centrifugal growth, displacing the adjacent nervous tissue rather than infiltrating it. In contrast, astrocytomas are known for their invasive and infiltrative nature. This leads to a more irregular appearance, less defined borders, and an eccentric positioning within the spinal cord [19].

Astrocytomas typically manifest as primarily solid masses, which may present either as entirely solid or as solid with areas of necrotic-cystic degeneration. According to prior studies, completely solid masses are observed in around 40% of astrocytoma cases [17, 20]. Our findings mirror this trend, with nearly 50% of astrocytic tumors in our study being completely solid, distinguishing them from ependymomas. In line with previous literature, we observed that the solid components of astrocytic tumors in our study tend to be hyperintense on T2WI [17].

Syringomyelia has been reported to be useful in differentiating ependymoma from astrocytoma independently [21]. However, in our findings, this distinction was not observed. Instead, syringomyelia was significantly more prevalent in low-grade PSGs and low-grade astrocytomas. Syringomyelia likely results from an obstruction in the normal flow of CSF and is typically observed in chronic and relatively benign conditions [18, 22]. We suggest that the chronic nature of low-grade tumors may contribute to the development of syringomyelia, in contrast to the rapid tissue infiltration observed in high-grade gliomas (HGG). Similarly, Chen et al. [18] reported that syringomyelia occurs more frequently in H3 K27 wild-type than in H3 K27M-mutant variants, a difference particularly notable in histological grade 2 astrocytomas.

Cysts were found to be associated with the tumor grade in PSGs and astrocytomas. Generally, cysts are acknowledged as a frequent characteristic of PAs [23]. Similarly, Kobayashi et al. [24] described cysts as a common feature in a series of WHO 2 Spinal ependymomas.

Identifying spinal cord-associated edema and its extension is crucial for accurate diagnosis and treatment planning. However, differentiating edema from a non-enhancing tumor in the spinal cord can be challenging, and sometimes not always possible. We observed that although on T2WI, both edema and tumor can appear hyperintense, edema generally has a more diffuse, widespread appearance. In contrast, non-enhancing tumors may show a more localized, mass-like effect. Similarly, on T1WI, while non-enhancing tumors generally remain isointense or hypointense, edema might also appear hypointense but is usually more spread out and less defined than tumor tissue. Like Crawford et al. [13], we found no significant correlation between edema and tumor grade, but this tended to be less common in low-grade gliomas (LGG). However, this difference was significant when analyzing tumor grade and astrocytomas alone. Edema occurs due to the breakdown of the blood-spinal cord barrier (BSCB), which is relatively intact in LGG and damaged in high-grade gliomas (HGG). Also, compression causes ischemia to cord vessels and swelling of astrocytes, causing cytotoxic edema. [25]. We argue that BSCB damage and direct involvement of the astrocytic cells might explain this difference.

Tumor enhancement is known as a valuable imaging biomarker reflecting the compromised integrity of BSCB.[9]. We found a statistically significant association between histological tumor grade and contrast enhancement patterns, where LGG were more likely to present a type 1A enhancement pattern and less likely to show a type 1B pattern. However, some authors have described that enhancement patterns found in HGG are not different from those in LGGs [9, 26]. This could be explained by the diversity of enhancing patterns associated with PAs. [23]. Seo et al. [9] suggested that patchy or irregular enhancement patterns are one of the MR imaging characteristics of astrocytomas in general, differentiating them from ependymoma which typically exhibits well-defined intense enhancement, with homogeneous (75%), heterogeneous, rim, or nodular pattern [9, 27]. However, we did not find this difference, as all tumors in this study showed a heterogenous enhancement pattern.

Kulkarni et al. [26], in a small report of spinal cord high-grade astrocytomas, revealed two predominant patterns of enhancement: rim enhancement and central inhomogeneous enhancement. Similarly, Crawford et al. [13] found that ring enhancement, though not statistically significant, was more typical of higher-grade lesions. Although there is no clear information on the margin appearance in either of these reports, these enhancement patterns could be extrapolated into our type 1b with central non-enhancement.

In our study, the association between leptomeningeal dissemination and tumor grade was statistically significant, with leptomeningeal dissemination being more common in HGG. Leptomeningeal dissemination of primary CNS tumors in children has been reported in HGG [26, 28, 29]. MYCN-amplified ependymomas for example, are aggressive tumors, with an increased likelihood of recurrence and metastasis, and often have leptomeningeal metastases at presentation [16, 30]. Although rare, leptomeningeal dissemination of spinal cord PAs has also been reported [28, 29, 31]. Two PAs in our study presented with leptomeningeal dissemination.

We found that, when only astrocytomas were taken into consideration, spinal canal widening was associated with tumor grade, being more common in low-grade astrocytomas. However, this was not true when analyzing all intramedullary PSG with tumor grade. Although not statistically significant, scoliosis was a common feature among low-grade astrocytomas. In our study, when scoliosis was present, the patient also had a spinal canal widening with an underlying low-grade astrocytoma. We did not see this feature in ependymomas, although it is estimated that 20–33% of patients with intramedullary spinal cord tumors present with concomitant scoliosis including both astrocytomas and ependymomas [11]. Scoliosis results from muscular imbalance, and asymmetrical weakness due to the tumor’s effect on trunk musculature [11, 32]. Pain and spinal rigidity are also considered partly responsible for scoliosis [33].

This study has some limitations, including the small number of cases due to the rarity of spinal cord astrocytomas and the use of variable MR imaging systems, ranging between 1.5T and 3T over twenty years.

## Conclusion

Based on our data, pediatric spinal gliomas exhibit several overlapping imaging characteristics, complicating the differentiation of tumor grades solely through imaging. Our findings suggests that the newly proposed enhancement pattern classification, might be a useful tool in distinguishing between high- and low-grade PSGs. Low-grade PSGs were significantly more likely to exhibit crisp, well-defined, smooth enhancing margins (type 1A enhancement pattern) and less likely to exhibit shaggy, irregular, ill-defined enhancing margins (type 1B enhancement pattern). We also found a higher frequency of cysts and syringomyelia in low-grade gliomas, while leptomeningeal enhancement is less common. Additionally, spinal canal widening is a prevalent feature in low-grade astrocytomas. Further research involving a larger sample size and multiple sites may be useful and necessary to assess the validity of this new classification system. Moreover, pilocytic astrocytomas, known for their diverse imaging characteristics, may sometimes resemble high-grade gliomas in imaging studies.
